# Supplementary material for: What helps and hinders reproducible research? Researchers’ perspectives from a cross-disciplinary interview study
Source: PLoS One. 2026 May 20;21(5):e0348512. doi: 10.1371/journal.pone.0348512 (PMC13189303; doi:10.1371/journal.pone.0348512)
Supplement: S2 Appendix — (PDF) [file pone.0348512.s002.pdf]

Reprinted from https://osiris4r.eu/media-center/ under a CC BY license, with permission from OSIRIS project Communications Manager, original copyright 2023.

**Appendix 2.**

**The recruitment strategy applied in this study is described in detail in the protocol published on Open Science Framework (open access):**

Kozula M, Van den Eynden V, DeVito NJ, Onghena P, Dudda L. Researchers' views, motivations, practices and barriers regarding reproducibility in research: Interview protocol. 2024. doi: 10.17605/OSF.IO/BT56A.
