# Supplementary material for: What helps and hinders reproducible research? Researchers’ perspectives from a cross-disciplinary interview study
Source: PLoS One. 2026 May 20;21(5):e0348512. doi: 10.1371/journal.pone.0348512 (PMC13189303; doi:10.1371/journal.pone.0348512)
Supplement: S3 Appendix — (PDF) [file pone.0348512.s003.pdf]

Reprinted from https://osiris4r.eu/media-center/ under a CC BY license, with permission from OSIRIS project Communications Manager, original copyright 2023.

INTERVIEW GUIDE: SEMI-STRUCTURED INTERVIEWS WITH RESEARCHERS

WP2 OSIRIS Open Science to Increase Reproducibility in Science

Creating trust in Open Science & Reproducibility through Accessibility and Transparency!

Funded by the European Union. Views and opinions expressed are however those of the author(s) only and do not necessarily reflect those of the European Union or the European Research Executive Agency (ERA). Neither the European Union nor the ERA can be held responsible for them.

Reprinted from https://osiris4r.eu/media-center/ under a CC BY license, with permission from OSIRIS project Communications Manager, original copyright 2023.

Keep in mind the aim & objectives of these interviews:

- Examine the researcher's views, motivations, personal practices, and barriers regarding reproducibility
- What reproducibility means in their research and how they personally view it
- Their experiences within their own research and within their field and science in general
- How they execute reproducibility
- Understand drivers, barriers & facilitators for reproducibility in the researchers' context

INTRODUCTION

Hi, thank you for being willing to help with our research and for taking the time to speak with me today. We have chosen you because we are interested in your views and experiences with reproducibility in research.

There are no right or wrong answers. However, please let me know if you don't want to answer a question or have no opinion on a matter. I will audio/video record this interview while we talk on Microsoft Teams. You can tell me to turn off the recording at any time. Do you have any questions at this point? You can ask questions at any point during the interview.

Okay, let's start. I will be making some notes while talking to you. I will now begin recording.

[Today is … My name is …]

Funded by the European Union. Views and opinions expressed are however those of the author(s) only and do not necessarily reflect those of the European Union or the European Research Executive Agency (ERA). Neither the European Union nor the ERA can be held responsible for them.

Reprinted from https://osiris4r.eu/media-center/ under a CC BY license, with permission from OSIRIS project Communications Manager, original copyright 2023.

BACKGROUND INFORMATION

Discussion topics:

- ✓ Professional background & research activity [Opening Line: Invite interviewee to talk briefly but freely]
- ✓ Employment history
- ✓ Type of research
- ✓ Publishing research results
- ✓ Research funding
- ✓ Engagement in collaborations in research projects

PROBES TO CLARIFY IF NEEDED

- Discipline
- Seniority level
- Gender

REPRODUCIBILITY FROM INTERVIEEWS' PERSPECTIVE: DEFINITION, PRACTICES AND IMPORTANCE

1. Topic: Defining reproducibility

- ✓ The meaning of reproducibility to the interviewee/the interviewee's definition of reproducibility.
- ✓ The definition/understanding of reproducibility in the interviewee's field.
  - If needed, clarify the reproducibility-replicability concepts with interviewee.
- ✓ Main factors influencing the reproducibility in the interviewee's field.

SCENARIO PROBES

- ✓ The experience of having the interviewee's research being reproduced.
  - Explore the factors that influenced the success/failure of this attempt (also: subject-specific factors/funding/infrastructure, etc.).
- ✓ The experience of reproducing someone else's work.
  - Explore the factors that influenced the success/failure of this attempt (also: subject-specific factors/funding/infrastructure, etc.).

REPHRASING

- Concepts corresponding with reproducibility: reliability, validity, trustworthiness
  - Criteria for reliability, validity, trustworthiness of research in the interviewee's research and in their research field
  - Any universally important criteria in all fields of science
  - Ways of increasing confidence in the research findings and conclusions
- A concept of someone wanting to confirm the interviewee's work: is it reproducible?
  - Type of information from the interviewee's typical study that needs to be shared to make their work reproducible
  - What would be needed for your research to be reproducible? What if one of your colleagues or a journal reader would like to reproduce your work?

Funded by the European Union. Views and opinions expressed are however those of the author(s) only and do not necessarily reflect those of the European Union or the European Research Executive Agency (ERA). Neither the European Union nor the ERA can be held responsible for them.

Reprinted from https://osiris4r.eu/media-center/ under a CC BY license, with permission from OSIRIS project Communications Manager, original copyright 2023.

2. Topic: Application of practices that aim to improve reproducibility

- ✓ Interviewee's practices that aim to improve reproducibility.
  - Explore the reasons for applying/not applying concrete practices.
- ✓ Interviewee's research routine and place of practices that aim to improve reproducibility.
- ✓ Interviewee's attitude towards practices that aim to improve reproducibility.
  - Explore: engage/avoid/neutral.
- ✓ Things the interviewee could do to make their research (more) reproducible.
- ✓ Practices that play a crucial role in increasing reproducibility in the interviewee's opinion.
  - Explore the interviewee's opinion on Open Science practices.

REPHRASING AND SCENARIO PROBES

- The essential aspects of the interviewee's research cycle to ensure reliable, trustworthy work.
- Ways for exploring the reproducibility practices
  - Early career researchers: training/supervisor instructions
  - Senior researchers: workflow in their lab/running the research team

3. Topic: Importance of reproducibility

- ✓ The importance level of reproducibility in the interviewee's research field.
  - Explore the reasons for this answer.
- ✓ The importance level of reproducibility for an interviewee and their research.
  - Explore the reasons for this answer.
- ✓ Responsibility to ensure research is reproducible.
- ✓ Irreproducible or unreplicable studies as threats to science.

PROBES

- Reproducibility/reliability, validity, trustworthiness of research as a topic:
  - In departmental and research team discussions
  - In debates in the interviewee's field
- Does science need to be reproducible/reliable, in the interviewee's opinion?

Funded by the European Union. Views and opinions expressed are however those of the author(s) only and do not necessarily reflect those of the European Union or the European Research Executive Agency (ERA). Neither the European Union nor the ERA can be held responsible for them.

Reprinted from https://osiris4r.eu/media-center/ under a CC BY license, with permission from OSIRIS project Communications Manager, original copyright 2023.

DRIVERS AND ENABLERS FOR REPRODUCIBILITY

1. Topic: Factors motivating interviewee to apply practices that aim at improving reproducibility

- ✓ Interviewee's motivations (drivers and reasons) to apply practices that aim at improving reproducibility.
  - Explore which play a crucial role for the interviewee and why.
- ✓ Institutional or environmental incentives and enablers that contribute to the interviewee's decisions on applying concrete practices.
  - Departmental/Institutional-level, National Policy-level, Stakeholders-level.
- ✓ Enablers/incentives not in place that would support the interviewee in applying practices that aim at improving reproducibility.
  - Departmental/Institutional-level, National Policy-level, Stakeholders-level.
- ✓ Incentives that don't influence the interviewee's decision to apply practices.

PROBES

- Reasons for not applying practices that aim at improving reproducibility.
- Factors that would motivate interviewee to apply practices that aim at improving reproducibility.
- Incentives and enablers to explore:
  - Journal/policy/learned societies' recommendations regarding reproducibility
  - Training on reproducibility
  - Rewards/promotion systems

Funded by the European Union. Views and opinions expressed are however those of the author(s) only and do not necessarily reflect those of the European Union or the European Research Executive Agency (ERA). Neither the European Union nor the ERA can be held responsible for them.

Reprinted from https://osiris4r.eu/media-center/ under a CC BY license, with permission from OSIRIS project Communications Manager, original copyright 2023.

BARRIERS AND FACTORS HINDERING ENGAGEMENT IN REPRODUCIBILITY

1. Topic: Barriers to applying practices that aim at improving reproducibility

- ✓ Challenges or difficulties with applying practices that aim at improving reproducibility in the interviewee's research work.
  - Field-specific barriers.
  - Explore if any particular barriers in interviewee setting/research work.
- ✓ Reasons for being discouraged/not being discouraged by the barriers.

2. Topic: Addressing the barriers

- ✓ Interviewee attempts/strategies to overcome barriers.
- ✓ Other ways to address these barriers that the interviewee could think of.
  - Any incentives or enablers that could help overcome these barriers.

PROBES

- Specific barriers or factors as reasons for not engaging in practices that aim at improving reproducibility in the interviewee's research work.
- X and Y barriers mentioned before in the conversation: how these can be addressed?

- ✓ **Closing question:** Is there anything else you would like to share with me regarding the topics of our conversation?

Funded by the European Union. Views and opinions expressed are however those of the author(s) only and do not necessarily reflect those of the European Union or the European Research Executive Agency (ERA). Neither the European Union nor the ERA can be held responsible for them.

Reprinted from https://osiris4r.eu/media-center/ under a CC BY license, with permission from OSIRIS project Communications Manager, original copyright 2023.

Appendix

If an interviewee for whom reproducibility is not relevant finds it challenging to discuss reproducibility guided by questions, provide a brief explanation of what the EU understands with reproducibility based on reports from 2020 and 2022 and explore whether how reproducibility is framed there means something for the interviewee in their research.

Reproducibility definitions

EU 2022 report Assessing the reproducibility of research results

Reproducibility is understood as a continuum from the reproduction of results (based on the same data, code and methodology) to replication (meaning the re-enactment/repeating of results using the same analytical method, but with different datasets). These processes rest on the availability of the data and methods from the original study. Thus, reproducibility relates to confirming the findings of the research (albeit with certain variations) in a similar setting.

EU report 2020 Reproducibility of scientific results in the EU

We consider reproducibility as a continuum based on three main research processes: reproduction, replication, and re-use. We use the term 'reproduction' (and reproducibility stricto sensu) to refer to the re-enactment of a study by a third party, using the original set-up, data and methodology of analysis (e.g. for certification). We use 'replication' for more general re-enactment of the results, using the same analytical method, but on different datasets (e.g. for comparison). And we use 're-use' for the more loose possibility to re-use the results beyond the original research context, both inside and outside the original scientific discipline (e.g. also for innovation, for transfer, for transdisciplinary research).

Funded by the European Union. Views and opinions expressed are however those of the author(s) only and do not necessarily reflect those of the European Union or the European Research Executive Agency (ERA). Neither the European Union nor the ERA can be held responsible for them.
