# Supplementary material for: What helps and hinders reproducible research? Researchers’ perspectives from a cross-disciplinary interview study
Source: PLoS One. 2026 May 20;21(5):e0348512. doi: 10.1371/journal.pone.0348512 (PMC13189303; doi:10.1371/journal.pone.0348512)
Supplement: S4 Appendix — (PDF) [file pone.0348512.s004.pdf]

Appendix 4.

Interviews with researchers OSIRIS

Codes\\Version 4 Final Themes Barriers and Facilitators

Name	Description
Barriers	
Theme 1. Navigating the Research Ecosystem	
I. Publishers	
1) Editorial Priorities	Editorial policies or decisions that do not support or prioritize reproducibility.
2) Lack of Journal Policy	Absence of journal requirements for reproducibility or related areas.
3) Peer Review	Issues with the peer review process in supporting reproducibility.
II. Institutions	
1) Lack of Institutional Policy, incl. rewards	Absence of institutional policies supporting reproducibility, including reward systems for making research reproducible.

Name	Description
2) Lack of Sustainability	No focus on continuity in exploring research problems as a result of "fast science" and the nature of work, which consists of publications.
3) Lack of Technical Support	Insufficient institutional backing for reproducibility efforts. Includes scenarios where there is insufficient guidance on existing resources; resources exist but are not signposted or incentivized.
4) Publish or Perish Culture	The focus on quantity of publications rather than quality of research work.
III. Funders	
Lack of Funders Policy and Follow-up Checks	Absence or limited funder policies on reproducibility or poor implementation, where policies exist.
Theme 2. Social and Cultural Dynamics as Drivers and Barriers	
1) Research Culture	Socially embedded norms and expectations among the research community that negatively influence the routine research practice in terms of reproducibility.
2) Ownership of Research and Data	Concerns about credit, priority and control that influence willingness and ability to share research materials.
3) Collaboration	Ways in which collaborative settings limit routines for documentation, sharing, and verification linked to reproducibility.
Theme 3. Resourcing Reproducibility	
1) Research Skills and Training	Lack of skills and/or training needed for reproducibility.

Name	Description
2) Infrastructure Issues	Lack of infrastructure or access to infrastructure needed for reproducibility practices, or infrastructure not meeting the specific research needs.
3) Cost and Lack of Funds	Financial barriers to making research reproducible.
4) Time and Competing Job Priorities	Time constraints that limit efforts to ensure reproducibility.
5) Resource Inequalities	Disparities in access to resources needed for reproducibility.
Theme 4. Inside the Research Process	
I. Contextual and Disciplinary Specificities	
1) Field- or Method-specific Barriers	Unique reproducibility challenges that are specific to a particular field. Field-specific does not strictly mean the challenge or characteristic is unique only to one discipline or research area.
2) Language Issues	Different aspects of barriers related to language in research.
II. Research Practices	
1) Data Access & Issues	Various problems with data access (such as paywalls, physical collections, data not shared), or when shared data is of poor quality.
2) Poor Specification (methods)	Insufficient detail in describing methods.
III. Sharing Constraints	Coming from external aspects

Name	Description
1) Data Privacy or Confidentiality	Challenges in ensuring reproducibility while protecting sensitive information.
2) Industrial Collaboration	Complications arising from collaborations with industry, where proprietary concerns may limit sharing.
Theme V. From Personal Commitment to Shared Responsibility	
No Barriers & Ready to Share	Participants reporting no barriers related to reproducibility-related practices.
Facilitators	
Theme 1. Navigating the Research Ecosystem	
1) Publishers	Journal policies and editorial practices that promote transparent and reproducible research.
2) Institutions	Policies, structures, and initiatives within research institutions that support and/or mandate reproducible research practices.
3) Funders	Policies and mandates set by research funders that encourage and/or enforce reproducibility.
Theme 2. Social and Cultural Dynamics as Drivers and Barriers	
1) Research Culture	Socially embedded norms and expectations among the research community that positively shape/influence the routine research practice in terms of reproducibility.

Name	Description
2) Collaboration	Ways in which collaborative settings foster routines for documentation, sharing, and verification linked to reproducibility.
3) Industry Impact	The importance placed on reproducibility for its applicability in industry or commercial contexts, driving researchers to meet external market or collaborative standards.
Theme 3. Resourcing Reproducibility	
1) Skills	Practical abilities of researchers needed in order to implement reproducible research practices throughout the research process.
2) Tools/Infrastructure	The access researchers have to software, platforms, and infrastructure that support reproducible research practices.
3) Guidelines, Standards, Manuals	Various guidelines that support reproducible research practices, such as standardized data or measurements or taxonomy recognized in the given field, reporting standards and checklists, field-specific guidelines, methods standardization, software/user manuals.
4) Awareness	General awareness-building about reproducible research among researchers.
Theme 4. Inside the Research Process	
1) Open Science Practices	Practices that make research more transparent, accessible, reusable, and reproducible across the entire research lifecycle.
2) Other specific for discipline or type of study	Other types of practices supporting reproducibility relevant to the field, discipline, or study-specific.

Name	Description
Theme 5. From Personal Commitment to Shared Responsibility	
I. Reflective Motivation	
1) Community Values & Service to Society	Community-wide values facilitating the reproducibility behaviour of the interviewee. Communality refers to the principle that scientific knowledge should be treated as a public good to be shared openly and freely among researchers and society.
2) Intrinsic Motivation	Personal commitment to quality in science, driven by honesty, curiosity, and responsibility. These values refer to moral and epistemological considerations about truth, objectivity, correctness, and various other scientific ideals.
3) Visibility and Reputation	Motivation to adopt transparent and reproducible workflows because doing so enhances a researcher's visibility, credibility, and professional standing.
II. Automatic Motivation	
1) Efficiency	The internal benefit of saving time and resources by employing methods that allow for easier re-analysis, reuse, and collaboration.
2) Fear, Uncertainty, or Doubt	Affective and cognitive hesitation about the adequacy of one's own documentation, data, code, or methods for external scrutiny or reuse.
3) Negative Experiences	Negative experiences facilitating reproducibility behaviour of interviewee.
4) Reactive-Passive	Reproducibility practices of a researcher facilitated by external requirements, such as journals or funders.

Name	Description
5) Validation	Ensuring the integrity, reliability, and credibility of research by enabling independent verification of methods and results. Verification of work aspects.
III. Improvements and Accountability	
Stakeholder Accountability	Statements assigning responsibility for ensuring reproducibility to specific stakeholders.
