# Supplementary material for: What helps and hinders reproducible research? Researchers’ perspectives from a cross-disciplinary interview study
Source: PLoS One. 2026 May 20;21(5):e0348512. doi: 10.1371/journal.pone.0348512 (PMC13189303; doi:10.1371/journal.pone.0348512)
Supplement: S5 Appendix — (PDF) [file pone.0348512.s005.pdf]

Appendix 5.

Qualitative Research Quality Strategies. Adopted from Lincoln and Guba's (1985) quality criteria for qualitative research.

Criterion	Strategy	Definition
Credibility	Peer debriefing	Regular debriefing sessions with key members of the research team.
	Pilot testing of the interview guide	Ensuring that questions are clear, relevant, and appropriate for the participants.
	Tailored probes and real-time member checks	Including probes and prompts tailored to participants, staying reactive, and using the ARC (ask, record, and confirm) technique (Zairul, 2021).
Transferability	Thick description	Including contextual and detailed descriptions in the analysis to allow others to assess the transferability of findings. Providing thick descriptions about the methodology.
Dependability	Audit trail	Maintaining transparency through detailed documentation of research procedures. Publishing a study protocol (Kozula et al., 2024).
	Stepwise replication of the data	Measuring inter-coder reliability (ICR) and inter-coder agreement (ICA) (Campbell et al., 2013).
	Proof of the analytical and interpretation process	Providing the codebook tree with definitions (Appendix 3).
	Raw data	Publishing interview transcripts (Kozula et al., 2025).
Confirmability	Triangulation: Investigator triangulation	Involving multiple researchers in the study design, data collection, codebook development, and analytical decisions to enhance reliability.

	Reflexive practice	Engaging in continuous reflection on researcher-participant dynamics, interpretive biases, and theoretical grounding (Caloran, 2003; Russell & Kelly, 2002). Providing positionality statements (Appendix 3).

References

Campbell, J. L., Quincy, C., Osserman, J., & Pedersen, O. K. (2013). Coding in-depth semistructured interviews: Problems of unitization and intercoder reliability and agreement. *Sociological Methods & Research, 42*(3), 294–320. https://doi.org/10.1177/0049124113500475

Carolan, M. (2003). Reflexivity: A personal journey during data collection. *Nurse Researcher, 10*(3), 7–14. https://doi.org/10.7748/nr2003.04.10.3.7.c5892

Kozula, M., Van den Eynden, V., DeVito, N. J., Onghena, P., & Dudda, L. (2024). *Researchers' views, motivations, practices and barriers regarding reproducibility in research: Interview protocol*. OSF. https://doi.org/10.17605/OSF.IO/BT56A

Kozula, M., DeVito, N. J., Onghena, P., Colombo, C., Muñoz Teno, P., & Van den Eynden, V. (2025). *Interviews with European researchers on open science and reproducibility in research, 2023–2024* [Dataset]. KU Leuven Research Data Repository. https://doi.org/10.48804/FMK8FK

Lincoln, Y. S., & Guba, E. G. (1985). *Naturalistic inquiry*. Sage.

Russell, G. M., & Kelly, N. H. (2002). Research as interacting dialogic processes: Implications for reflexivity. *Forum Qualitative Sozialforschung / Forum: Qualitative Social Research, 3*(3), Article 18. https://doi.org/10.17169/fqs-3.3.831

Zairul, M. (2021). Can member check be verified in real time? Introducing ARC (Asking, Record, Confirm) for member checking validation strategy in qualitative research. *Engineering Journal, 25*(1), 245–251. https://doi.org/10.4186/ej.2021.25.1.245

Positionality statements of the core research team

Magdalena Kozula

I am a qualitative researcher trained in international and higher education studies, with professional experience across higher education policy and academic settings. My scholarly interests developed over the years of working on academic policies, including roles in universities, governmental bodies, and the third sector across Europe and the US. These experiences, along with my training in intercultural communication, helped me cultivate reflexivity about my own assumptions and provided me with resources that help me deepen my understanding of diverse academic contexts.

As a white woman from Central Eastern Europe, I often occupy an 'in-between' position across cultural frames - familiar enough to relate, distant enough to invite explanation. I aim to meet interlocutors on their own terms. I see qualitative methods as essential for making visible the situated, sometimes conflicting logics. In this research, I view myself as an active instrument in the research process, and my resources as assets that can enhance, rather than undermine, the study, especially when a researcher speaks with another researcher about research. Having said that, I acknowledge my biases as sympathetic to open science, and I separate description from advocacy by letting participants' accounts lead, continually interrogating my assumptions, and documenting analytic decisions to preserve transparency throughout the study.

Nicholas DeVito

I am a cis-gendered white male from a Western background. My training is in the social study of science and medicine and in public health and science policy, often utilizing a mixed-methods framework. I am interested in the ways qualitative research can complement and explain quantitative findings. As a researcher, I do not come to questions of open science and reproducibility from the position of a neutral, naive observer. While I am quite cognizant of where equipoise exists within the field, and can acknowledge the many limitations of existing practice and evidence, I am also a practitioner, teacher, and advocate for open science practices, and ultimately would hypothesize that they do tend to improve reproducibility, with minimal or manageable negative externalities. While I make every effort to remain cognizant of this potential bias in my conduct and interpretation, I also believe it is important to acknowledge this when acting as a researcher of the practices and phenomena within this space.

Patrick Onghena

I was trained as a postpositivist researcher and my approach is still mainly postpositivist. My way of doing science is mainly from a realist perspective. This means that I'm mostly interested in replicability of phenomena and processes in a world that exists independently of our perception and interpretation. Because we have no direct access to these phenomena and processes, we have to rely on indicators, fallible measurements, interpretation, and intersubjective control and verification.

I consider theory, conceptual analysis, and empirical work to be of equal importance for scientific progress. In my empirical work, I like to combine quantitative and qualitative material, quantitative and qualitative analysis, inferential statistics and interpretation. In my collaborations with other researchers, I'm usually taking a flexible methodological stance and looking for approaches, ways of data collection and data analysis that best fit the problem and research questions at hand. However, in terms of philosophical foundation, I always tend in the direction of Peirce's pragmatism, which is focused on clarifying concepts through their observable consequences and exploring their implications for informed practice.

Cinzia Colombo

I am a white woman from a South European background, trained in philosophy, with a focus on philosophy of science. I have been working as a researcher in the field of citizen involvement in research for the last 22 years. Before that, I worked in the field of scientific journalism in biomedicine and environmental epidemiology. I volunteer in the field of healthcare and advocacy for the rights of people without a residence permit, refugees, Roma, and Sinti. My training, work experience and voluntary activity have fostered my interest in the critical appraisal of knowledge and knowledge production. In my research activities, I refer to participatory approaches. I usually worked in a quantitative research environment, then I realised the need to train myself in qualitative research.

My interest in research reproducibility arose from an interest in open science, from both a practical and epistemological point of view. In this research, I am an insider of the research community, being a researcher myself, but I also feel that I have an outside perspective due to my training and background.

Veerle Van den Eynden

I am a white female from a Western European background, trained in agricultural and applied biological sciences. My research - 16 years on people-plant and people-environment interactions, then 18 years on research data management and open science - uses mixed methods and participatory techniques. I acquired social science and anthropology methods skills through doctoral training and self-learning. Significant parts of my research career I did grassroots research and lived in the Global South, where I experienced firsthand the benefits of data sharing, and the restraints to research through lack of access to data and scientific literature. It also gave me a fascination for cultural and ethical differences. In my research, I take a pragmatic and rational stand, seek where evidence can be applied and implemented in practice, and write in plain language.

In this research on open science and reproducibility, I am an insider to the community studied (European researchers), and aware of my bias in favour of open science and transparency in qualitative research. Aware of this bias, during interviews and analysis, I keep a neutral view as an outsider to let researchers tell their own views and realities.
